# Resistance Training Beyond Momentary Failure: The Effects of Past‐Failure Partials Versus Initial Partials on Calf Muscle Hypertrophy Among a Resistance‐Trained Cohort

**DOI:** 10.1002/ejsc.70030

**Published:** 2025-08-24

**Authors:** Stian Larsen, Nordis Ø. Sandberg, Brad J. Schoenfeld, Andrea B. Fredriksen, Benjamin S. Kristiansen, Milo Wolf, Roland van den Tillaar, Paul A. Swinton, Hallvard N. Falch

**Affiliations:** ^1^ Department of Sports Science and Physical Education Nord University Levanger Norway; ^2^ Academy for Personal Training Fredrikstad Norway; ^3^ Department of Exercise Science and Recreation Applied Muscle Development Laboratory CUNY Lehman College Bronx New York USA; ^4^ School of Health Sciences Robert Gordon University Aberdeen UK

**Keywords:** calves, muscle length, muscle thickness, plantarflexion, ultrasonography

## Abstract

This study compared calf training with initial partial repetitions versus full range‐of‐motion (ROM) repetitions followed by past‐failure partials on gastrocnemius hypertrophy. Twenty‐three participants (men: *n* = 16 and women: *n* = 7) performed four sets of unilateral Smith machine calf raises to momentary failure twice a week for 8 weeks. One leg was trained using initial partials to their individualized maximum dorsiflexion ROM. The contralateral leg was trained with a full ROM and continued with past‐failure partials after failure in peak plantarflexion. Medial gastrocnemius muscle thickness was measured with ultrasonography both baseline and postintervention. A Bayesian framework was used to estimate the average treatment effect (ATE) using credible intervals and Bayes factors (BFs). The ATE posterior distribution indicated a greater increase in muscle hypertrophy for the initial partial condition (0.40 [95% CrI: −0.06 to 0.85 mm]; *p* (> 0) = 0.958), with a BF of 1.2 suggesting “anecdotal” evidence in favor of an effect. Within‐condition analyses using standardized mean difference estimates indicated that the interventions were likely to produce medium to large improvements. These findings suggest that both initial partials and past‐failure partials are viable strategies for achieving gastrocnemius hypertrophy. Although the average change favored initial partials, the estimated difference was uncertain, and the Bayes factor provided only anecdotal support for a differential effect.

## Highlights

- This study compared calf raises performed with initial partial range of motion (ROM) training to past‐failure partial ROM training for medial gastrocnemius hypertrophy.
- Both training conditions resulted in increases in muscle thickness following an 8‐week intervention among resistance‐trained individuals.
- Bayesian analysis provided anecdotal evidence in favor of initial partials; however, the credible intervals for group differences included zero, suggesting preliminary findings.
- Among resistance‐trained individuals aiming to optimize medial gastrocnemius hypertrophy, both strategies may be effective, with initial partials appearing to induce slightly greater growth.

## 1 Introduction

Resistance training is an effective strategy for inducing skeletal muscle hypertrophy in humans (Roberts et al. 2023). Numerous studies have examined the effects of resistance training variables, such as volume (Baz‐Valle et al. 2022), frequency (Schoenfeld et al. 2016), and intensity (Robinson et al. 2024), on the hypertrophic response. Another training variable that has garnered increased attention in recent years is range of motion (ROM) (Kassiano et al. 2022). In single‐joint exercises, such as the calf raise, ROM typically refers to the joint angular range of a movement (Coratella 2022). Moreover, ROM is typically categorized as either full ROM or partial ROM (Moreno et al. 2024). Partial ROM can be further categorized into shortened partials (i.e., performing the exercise through the ROM where the target muscle is in a shortened position) or lengthened/initial partials (i.e., performing the exercise through the ROM where the target muscle is in a lengthened position). Wolf et al. (2023) conducted a meta‐analysis on the effects of ROM on muscle hypertrophy and reported a trivial standardized mean difference favoring full ROM over partial ROM. Additionally, they reported a standardized mean difference of −0.28 (95% CI: −0.81 to 0.16) in favor of initial partials compared to full ROM training. Based on these results, the researchers concluded that training with both full ROM and initial partials may enhance muscle size outcomes.

As mechanical tension is considered a primary stimulus for muscle hypertrophy through mechanotransduction (Wackerhage et al. 2019), a recent review suggested that the length‐tension relationship may determine which muscles benefit the most from training at longer lengths (Ottinger et al. 2023). Several studies examining sarcomere length have reported that muscles may operate at different points on the length‐tension curve, including the ascending limb, plateau region, and descending limb, either in combination or separately (Cutts 1988, 1989; Son et al. 2018). Notably, the biarticular gastrocnemius has been observed to operate at the ascending limb (Maganaris 2003), which reduces its force production when it operates at a short muscle length (Maganaris 2003). This may explain why three recent independent studies have found that training the gastrocnemius at longer muscle lengths can result in greater hypertrophic adaptations compared to training at shorter muscle lengths (Burke et al. 2024; Kassiano et al. 2023; Kinoshita et al. 2023). For instance, Kassiano et al. (2023) examined gastrocnemius thickness changes when performing calf raises (knee extended) under three conditions: a full ROM (−25 dorsiflexion to +25° plantarflexion), a shortened partial ROM (0 to +25°), or an initial partial ROM (−25° to 0°). The authors reported the greatest medial gastrocnemius hypertrophy for the initial partial group, with no significant differences between groups for the lateral gastrocnemius.

Anecdotally, some practitioners have applied findings from the initial partials literature (Wolf et al. 2023) by incorporating past‐failure partials (Larsen et al. 2025). This strategy involves performing full‐ROM repetitions to volitional or momentary muscular failure at a shortened muscle length, followed by partial repetitions at longer muscle lengths to further failure (hereafter referred to as past‐failure partials). Past‐failure partials can be used to extend a set beyond what is typically considered the ultimate set termination point, with the aim of increasing intraset repetition volume and time spent when the muscle is in a more lengthened position. For example, our laboratory compared standing calf raises performed solely with full‐ROM repetitions to momentary failure near peak plantarflexion versus past‐failure partials assessing medial gastrocnemius muscle thickness (Larsen et al. 2025). We observed a 0.62 mm greater increase in medial gastrocnemius muscle thickness for the past‐failure leg. However, Kassiano et al. (2023) reported a 15.2% increase in medial gastrocnemius thickness following initial partial training, whereas we observed only 9.6% growth with past‐failure partials. Thus, in our previous study, we postulated that performing only initial partial repetitions during calf raises could be more effective than past‐failure partials for increasing medial gastrocnemius hypertrophy. However, differences between studies could also be due to sampling variance and numerous factors. Given the lack of direct comparisons between these two strategies, further investigation is warranted.

Therefore, the present study aimed to compare the effects of training exclusively with initial partials versus full‐ROM repetitions followed by past‐failure partials on gastrocnemius muscle thickness. Based on our previous speculations (Larsen et al. 2025), we hypothesized that initial partials would be superior to past‐failure partials for promoting calf muscle growth.

## 2 Methods

### 2.1 Participants

Sample size determination was based on previous studies by our group (Larsen et al. 2024, 2025) that manipulated ROM using a Bayesian and a within‐participant design. This framework was chosen as it enables the quantification of differences between conditions based on plausible values and the assessment of evidence strength against an a priori null hypothesis. A within‐participant design with priors was used to enhance estimation precision and control for genetic and lifestyle‐related factors (MacInnis et al. 2017).

**FIGURE 1:**
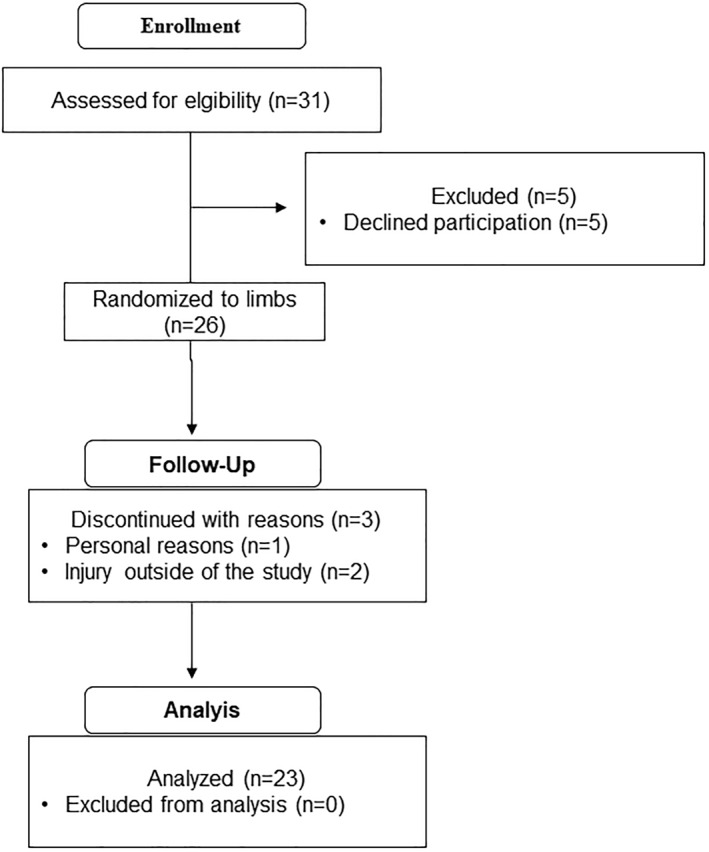
Consort diagram illustrating the data collection process. The enrollment process was similar to Larsen et al. (2025).

Simulation‐based calibrations using Bayes factors were conducted to assess whether plausible effect sizes given our constraints were sufficient to support the correct hypothesis with sample sizes of *n* = 25 and *n* = 30, respectively. Priors were derived from previous studies and meta‐analyses (Larsen et al. 2025; Swinton et al. 2022; Wolf et al. 2023). The priors set on a standardized scale included distributions for typical improvement *N* (0.44,0.40^2^), average treatment effect *N* (0.30,0.27^2^), heterogeneous response *N* (0,0.15^2^), and measurement error *N* (0,0.20^2^). Bayes factor calibrations were performed over 500 iterations, with an average treatment effect of zero (indicating no difference between interventions) in half of the simulations, whereas a nonzero distribution was applied in the other half. For sample sizes of *n* = 30 and *n* = 25, the average posterior model probability was 49.7% (95% CrI: 41.2% to 55.9%) and 48.6% (95% CrI: 40.0% to 56.2%), respectively. The proportion of posterior probability assigned to the alternative hypothesis when it was true was 84% and 81%, respectively, for the two sample sizes. Based on these findings, we concluded that this approach provided adequate evidential strength. Consequently, we aimed to recruit 30 participants, eventually including 26 (Figure 1 and Table 1). Twenty‐three participants completed the intervention (height: 174 ± 9.9 cm, age: 28.0 ± 5.6 years: Table 1). Participants reported a weekly calf training frequency of 1.1 ± 0.8 workouts (range: 0–2) and a weekly calf raise set volume of 4.3 ± 3.2 sets (range: 0–12) in the 12 months preceding the study. Three participants reported no weekly calf raise training during this period. All procedures were conducted in accordance with the latest version of the Declaration of Helsinki. The project received approval from SIKT (ref: 578814), and the study's ethical considerations were reviewed by REK, which determined that further ethical approval was not required (ref: 795724).

**TABLE 1:** Descriptive summary of participant characteristics.

	Men (*n* = 16)		Women (*n* = 7)	
Variables	Mean (SD)	Range	Mean (SD)	Range
Age (years)	29.3 ± 6.0	22–41	25.3 ± 3.6	21–32
Body mass (kg)	85.1 ± 14.6	—	68.1 ± 14.0	—
Height (cm)	178.6 ± 7.4	168–197	163.6 ± 6.5	160–173
Peak dorsiflexion (°) past‐failure partials	30.1 ± 6.4	23–44	31.3 ± 6.9	19–41
Peak dorsiflexion (°) initial partials	29.0 ± 6.0	23–41	31.3 ± 5.7	25–43
RT experience (years)	7.4 ± 4.0	3–16	7.2 ± 2.4	5–11
Weekly calf set volume	4.1 ± 3.2	0–12	5.0 ± 2.8	0–8
Weekly calf frequency	1.1 ± 0.8	0–2	1.3 ± 0.7	0–2

Participants met the following inclusion criteria: (1) age range between 18 and 50 years; (2) no previous self‐reported use of anabolic steroids or other muscle‐enhancing illegal agents; (3) resistance trained, defined as having performed at least two RT sessions a week for the last 3 years prior to the start of the intervention; and (4) no injury or illness that could compromise the results or participation of this study. However, participants were not required to have specific training experience for the calf muscles as this study was a part of a larger research project.

### 2.2 Risk of Confounding Variables and Bias

Prior to data collection, the study's aim, hypothesis, and methods were preregistered on the Open Science Framework (https://osf.io/f26u5). The study adhered to the Standards Method for Assessment of Resistance Training in Longitudinal Design (SMART‐LD) checklist (Schoenfeld et al. 2023) to minimize bias (see Supporting Information S1). The supervised training program included three exercises: calf raises, lateral raises, and leg presses, each addressing distinct research questions (Larsen et al. 2025a, 2025b). Details on the other research questions are available in the preregistrations (osf.io/zmkhw; osf.io/847ep). To reduce potential confounding, we standardized the number of sets, repetition range, plantarflexion ROM, proximity‐to‐failure, rest intervals, and lifting durations for both the eccentric and concentric phases. Each of these variables may influence the resistance training stimulus and causal interpretation (Coratella 2022).

### 2.3 Resistance Training Procedures

As a within‐participant design, the left and right limbs were randomized using www.randomizer.org, with allocation concealed from investigators and participants until baseline testing. Each limb was trained unilaterally to momentary failure using either initial partials (maximal dorsiflexion to a neutral) or past‐failure partials, consisting of a maximum of full repetitions (full ROM: maximal dorsiflexion to maxima; plantarflexion) followed by past‐failure initial partials. To recruit trained participants, the study included a full‐body resistance training program with additional randomized limb comparisons including the lateral raises (cable vs. dumbbell) and leg press (full vs. restricted ROM). This manuscript focuses exclusively on calf raises and medial gastrocnemius hypertrophy.

All participants performed unilateral straight leg calf raises in a Smith machine (GymSport AS, Trondheim, Norway) with an individualized ROM determined during a familiarization session. The exercise was executed on a step box with antiskid tape to prevent slipping. All calf raise training was instructed to be performed with the knee joint extended. During the first two sessions, participants completed three unilateral sets of standing Smith‐machine calf raises for each limb, totaling six sets in the first week. For the subsequent 7 weeks, participants completed four unilateral sets per limb, twice weekly (eight sets per week). Prior to the intervention, participants attended a familiarization session to determine their 20‐repetition maximum (20RM) for both variations of the standing calf raise.

**FIGURE 2:**
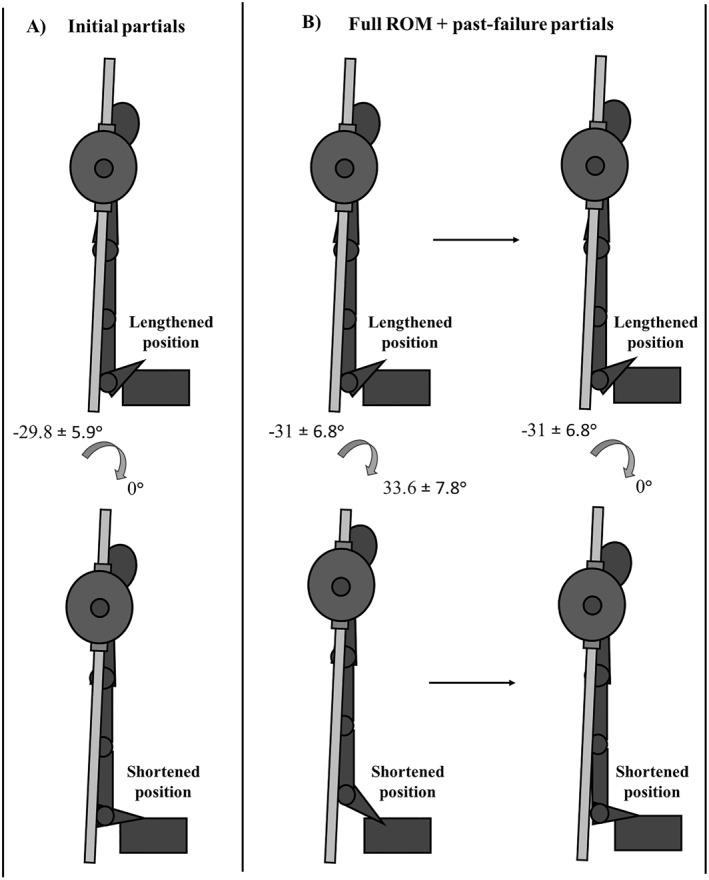
Illustrates the plantarflexion ROM for the (A) initial partials and (B) past‐failure partial legs, respectively.

The repetition range was set at 10–20 RM for the initial partials and 5–10 + 5–10 RM for the past‐failure partials to ensure similar repetition volumes across conditions. The initial partial condition performed calf raises for an individualized partial ROM (−29.8 ± 5.9° to 0°), where 0° was defined as a 90° angle between the tibia and the foot (Kassiano et al. 2023). For the past‐failure partials condition, participants performed full ROM calf raises between their individualized peak dorsiflexion and peak plantarflexion ROM (−31 ± 6.8° to +33.6 ± 7.8°) and continued with only past‐failure partials (−31 ± 6.8° to 0°) after reaching momentary failure in plantarflexion (Figure 2). To measure ankle dorsiflexion and plantarflexion angles, an electric goniometer (Ease angle, Stockholm, Sweden) was used. For both groups, all sets were performed to momentary concentric failure, defined as failing to match the barbell height achieved on the first repetition. In the full‐ROM condition, momentary failure was determined when the participant could no longer reach the marked peak barbell height, after which participants continued with past‐failure partials. For past‐failure partials, momentary failure was defined as the inability to ascend to 0° of plantarflexion. The same definition was applied in the initial partial condition (Figure 2).

Load was adjusted throughout the intervention. If participants successfully completed more than 10 + 10 repetitions or more than 20 repetitions in a single set, the external load was increased by 0.25 kg. If participants failed to meet the repetition range threshold (< 5 + 5 or < 10 repetitions), the external load was decreased by 0.25 kg. Participants were instructed to maintain a tempo of approximately one second for both the eccentric and concentric phases. A slight pause was required in full dorsiflexion, but no pause was permitted in full plantarflexion or at the neutral position. Rest intervals were standardized, with approximately 30 s between training limbs and 90 s between sets for the same limb. To minimize the potential confounding effect of exercise order, the limb and condition that trained first varied weekly.

Participants were also provided an optional resistance training program, which included horizontally loaded pectoralis major exercises, Romanian deadlifts, seated rows with a narrow grip, elbow flexion, elbow extension, and lat pulldowns (see Supporting Information S1). No additional exercises targeting the triceps surae muscles were permitted during the intervention, including leg curls, Nordic hamstring curls, or any other calf raise variations. All training sessions were directly supervised by at least one research assistant. The supervision team consisted of individuals with at least a bachelor's degree in sports science, along with MScs and PhDs. Supervisors were given detailed instructions on the resistance training procedures before the start of the study by the lead researcher. Supervisors also participated in two pilot tests to ensure they were familiar with all aspects of the training procedures. The data collection and resistance training intervention were conducted at CARE Treningssenter Levanger (Levanger, Norway).

### 2.4 Nutritional Guidelines

Participants were advised to increase their caloric intake modestly and to consume at least 1.6 g of protein per kilogram of body weight daily as recommended by (Morton et al. 2018). Body mass was tracked weekly, with participants weighed at the beginning of each training week using a Tanita scale (MC‐780MA, Riga, Latvia).

### 2.5 Muscle Thickness Assessments

Muscle thickness was assessed using B‐mode ultrasonography (Echo Wave 2 Software; Telemed, Latvia) with a 60‐mm probe (9 MHz) and Chemolan transmission gel (Chemodis, DA, Alkmaar). Measurements were taken at two preintervention and postintervention time points to increase precision. Prior to ultrasound assessments, participants were required to refrain from resistance training or other physically demanding activities for a minimum of 72 h before the measurements. Additionally, they were instructed to avoid food for 2 hours and caffeine for 8 hours before their scheduled laboratory visit. The validity and reliability of ultrasound assessments for changes in muscle size have been reported to be high, including comparisons to the gold standard magnetic resonance imaging (Reeves et al. 2004).

Ultrasound imaging was performed longitudinally, with the probe positioned at the thickest and salient region of the medial gastrocnemius from a posteroanterior view (Kassiano et al. 2023). To ensure consistency, a pen was used to mark reference lines, and photographs of the markings were taken for each participant to replicate placement at postintervention. Images were stored on a password‐protected flash drive, with access restricted to the two examiners conducting the measurements. One examiner controlled the probe, whereas the other captured the images. Three images were taken from each leg, and an average of the preintervention and postintervention values was calculated. If one of the images deviated by more than 10% from the others, a fourth image was taken. In this study, the typical error and coefficient of variation for ultrasound measurements were below 0.36 mm and 1.6%, respectively.

## 3 Statistics

All statistical analyses were performed using R (version 4.4.0) within a Bayesian framework. To account for the repeated‐measures design inherent to the within‐participant approach (Magezi 2015), univariate linear mixed‐effects models were employed incorporating random effects. The primary focus of estimation was the average treatment effect (ATE), which represents the mean difference in muscle thickness change scores between the experimental conditions applied to each limb.

Inference was drawn from the posterior distributions of ATE estimates, with credible intervals used to quantify uncertainty. Additionally, Bayes factors (BFs) were calculated to evaluate the strength of evidence supporting a nonzero ATE (H_1_) compared to a null effect (H_0_). Standard qualitative classifications of BF values were applied to aid interpretation (Aczel et al. 2018).

The magnitude of within‐condition changes was also quantified to evaluate the overall effectiveness of each intervention independently and interpreted against thresholds specific to strength and conditioning (Swinton and Murphy 2022). A structured Bayesian workflow guided the analytical process. Informative priors were selected based on meta‐analyses relevant to resistance training interventions (Swinton and Murphy 2022), and prior predictive checks were conducted to ensure their suitability. The models underwent multiple iterations to assess the consistency of estimates, while posterior predictive checks and sensitivity analyses using non‐informative priors were carried out to test the robustness of findings. Furthermore, simulation‐based calibration of Bayes factors was implemented to refine the interpretation of evidential strength. To promote accuracy, reproducibility, and methodological rigor, the analysis adhered to the WAMBS checklist (When to Worry and How to Avoid Misuse of Bayesian Statistics). Summaries of the Bayesian workflow, including prior and posterior evaluations, are reported in Supporting Information S3.

## 4 Results

### 4.1 Attendance

Participants attended an average of 15.2 out of 16 resistance training sessions. Of the 26 participants initially enrolled, 23 completed the intervention and were included in the final analysis. Two participants withdrew due to injuries not related to the study, whereas one participant withdrew for personal reasons. No other adverse events occurred during the interventions.

### 4.2 Muscle Hypertrophy

**FIGURE 3:**
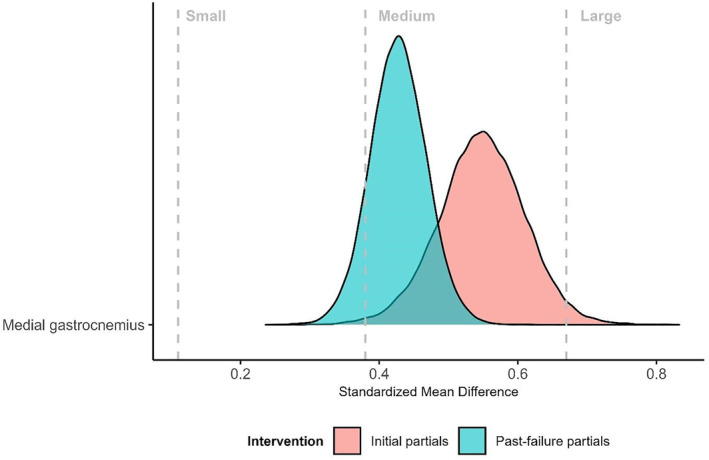
Comparative distribution plot of estimated standardized mean difference of interventions. Density plots illustrate estimates and uncertainty of standardized mean difference changes across the two interventions. Thresholds describing the magnitude of improvements are obtained from strength and conditioning‐specific data.

The posterior distribution indicated greater muscle hypertrophy in the initial partial condition with an estimated 0.40 mm increase (95% CrI: −0.06 to 0.85 mm) and a posterior probability of *p* (> 0) = 0.958. The Bayes factor (BF = 1.2) suggested “anecdotal” evidence in support of a beneficial effect. A subset analysis restricted to the 19 participants with prior calf‐specific resistance training experience in the 12 months leading up to the study, produced similar results, showing an increase of 0.47 (95% CrI:−0.04 to 0.95 mm), with *p* (> 0) = 0.967 and BF = 1.3. Within‐condition analyses using standardized mean difference estimates indicated that both training interventions were likely to produce medium to large improvements (Figure 3 and Table 2). The WAMBS checklist output and Bayes factor simulation‐based calibration, provided in the supplementary materials, identified no concerns regarding the analyses.

**TABLE 2:** Descriptive summaries of data (mean ± SD).

Outcome	Baseline initial partials	Post‐test initial partials	Δ%	Baseline past‐failure partials	Post‐test past‐failure partials	Δ%
Medial gastrocnemius (mm)	20.4 ± 3.3	22.3 ± 3.3	9.5 ± 4.3	20.7 ± 3.5	22.0 ± 3.6	6.7 ± 3.3

### 4.3 Volume Load

**FIGURE 4:**
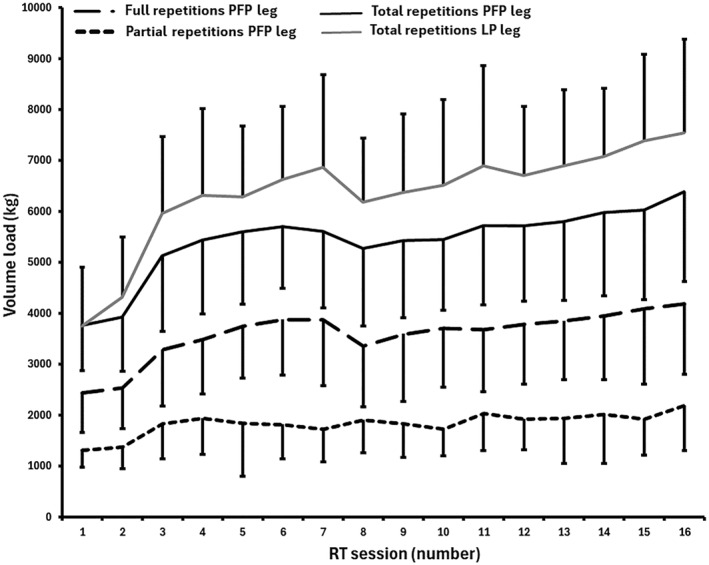
Mean (SD) volume load (sets × load × repetitions) lifted each resistance training session for the initial partials and past‐failure partials leg. LP = lengthened partials; PFP = past‐failure partials; RT = resistance training.

Total volume load increased as training progressed. In sessions 1 and 2, the past‐failure partial condition produced an average of 3763 ± 883 kg and 3925 ± 1061 kg, whereas the initial partial condition produced 3756 ± 1149 kg and 4323 ± 1181 kg, respectively. By session 3, when the weekly set volume increased from six to eight sets, total volume load increased to 5134 ± 1482 kg and 5964 ± 1503 kg, respectively. By the final training session, total volume load was 6391 ± 1768 kg and 7538 ± 1849 kg for the past‐failure partials and initial partials conditions, respectively (Figure 4). The average volume load from week 2 onward was 3757 ± 248 kg for full ROM repetitions and 1908 ± 120 kg for past‐failure partials, resulting in a combined total of 5664 ± 314 kg. In contrast, the lengthened partial condition exhibited a higher average volume load of 6686 ± 437 kg across all sessions from week 2 onward.

## 5 Discussion

The findings of this study suggest that initial partials may be more effective than past‐failure partials for promoting calf muscle hypertrophy, although the strength of evidence supporting this hypothesis was limited. Descriptively, both training interventions led to increases in medial gastrocnemius thickness, with average changes of 1.9 mm (9.5%) for initial partials and 1.4 mm (6.7%) for past‐failure partials. The estimated population‐level difference favored initial partials by 0.40 mm, but the 95% credible interval (−0.06 to 0.85 mm) included zero, indicating substantial uncertainty. Moreover, a specific test of our a priori hypothesis yielded only anecdotal evidence in favor of an effect (Bayes factor = 1.2), highlighting that although the effect may exist, stronger data are required to substantiate this claim.

These findings are consistent with those of Kassiano et al. (2023), who reported a 15.2% increase in medial gastrocnemius thickness for initial partials compared to 6.7% when using full‐ROM repetitions. Similarly, recent research from our laboratory found evidence supporting the effectiveness of calf raises performed to momentary failure in the most lengthened position (9.6%) compared to terminating the set at failure in the shortened position (6.7%) (Larsen et al. 2025). However, participants in our previous study were untrained, which may explain the larger training response, as prior training volume has been suggested to influence hypertrophic adaptations (Scarpelli et al. 2022). In contrast, participants in the current study reported an average weekly calf training set volume of 4.2 ± 3.1 sets (range: 0 to 12) prior to the study, which may be considered relatively low (Schoenfeld et al. 2017). Additionally, approximately half of the participants reported training the calf muscles in a seated position, which has been shown to be less effective for gastrocnemius hypertrophy than training with the knee extended (Burke et al. 2024; Kinoshita et al. 2023).

To further contextualize the present findings in relation to previous research (Kassiano et al. 2023) and address limitations of this study, we can consider plausible mechanistic explanations. Importantly, both training conditions in this study were equated for failure criteria—sets in both conditions ended when participants could no longer complete another partial repetition. Thus, observed differences in hypertrophy cannot be attributed to differences in proximity to failure, as might have been the case in our earlier study where sets were terminated at past failure (Larsen et al. 2025), but rather suggest an inherent benefit to training the gastrocnemius at longer muscle lengths.

Cunnane et al. (2023) demonstrated that the medial gastrocnemius operates on the ascending limb of the force‐length curve, where it generates significantly lower force in plantarflexed positions than in dorsiflexed positions. This implies that tension per unit of cross‐sectional area may be diminished when the muscle is trained in a shortened position (Jorgenson et al. 2020), potentially explaining why past‐failure partials, which involved more time at shorter muscle lengths resulted in less hypertrophy. In contrast, training at longer muscle lengths with initial partials may have produced greater mechanical tension, a key stimulus for muscle hypertrophy (Wackerhage et al. 2019).

Furthermore, Beck et al. (2022) found that contracting into peak plantarflexion, as required in the past‐failure partial condition, incurs higher metabolic cost without increasing mechanical work. Such differences in metabolic demand may have influenced fatigue accumulation and the point at which participants reached momentary failure. Given that the medial gastrocnemius functions on the ascending limb of the force‐length curve (Cunnane et al. 2023) and experiences greater metabolic demands in the past‐failure partials condition (Beck et al. 2022), these factors may help explain the greater volume load observed in the initial partials condition (Figure 4). This aligns with the findings of Kassiano et al. (2023), who similarly reported higher volume loads with initial partials compared to full‐ROM calf raises. However, our study expands on theirs by demonstrating that this difference in volume load was maintained throughout the entire intervention, even when partials were added after failure in the full‐ROM condition.

Although the findings indicate that initial partials may be superior to past‐failure partials for promoting calf hypertrophy, the strength of the evidence was limited. The estimated difference in mean muscle thickness change between conditions was 0.40 mm, with a high subjective probability that this value exceeded zero (*p* [> 0] = 0.958). When expressed as a percentage of the observed data, the results showed a 2.8% greater increase favoring initial partials. However, direct comparison of the predictive performance of the two hypotheses resulted in a Bayes factor of only 1.2, suggesting that the observed difference is best considered tentative and not strongly supported by the data.

## 6 Limitations

An important limitation of this study, shared with many resistance training studies, was its relatively short duration. A longer intervention may have provided greater clarity regarding potential differences between initial partials and past‐failure partials. Nonetheless, as typical training cycles last approximately 8 weeks, our findings still offer practical insights into how these strategies may be implemented in real‐world training programs. A key limitation is the combination of relatively high measurement error (0.36 mm) and limited evidential strength, which together reduce confidence in the observed effects and their generalizability. Further research with larger cohorts and longer study durations is needed to validate these findings and better assess their generalizability. Another limitation is that participants simultaneously trained unilateral leg press at two different knee flexion angles (100° vs. full ROM) as part of a separate study (Larsen et al. 2025). Although leg press variation was randomized across limbs, an increased knee flexion angle may correspond to an increased dorsiflexion angle and potentially a greater calf stimulus. Thus, some degree of confounding remains a possibility. Also, dietary intake was not tracked, although weekly weight monitoring confirmed that participants adhered to a caloric surplus. Although a within‐participant design was used, no nontraining control group was included in the study. This may have limited the ability to quantify measurement error over time (Hammert et al. 2024). In addition, although all participants had general experience with resistance training, some did not specifically train their calf muscles directly and would have thus only received ancillary stimulation of the triceps surae via multijoint lower body training. Finally, muscle thickness measurements were only taken at the medial head of the gastrocnemius, which may be more responsive to training at longer muscle lengths. Future research should aim to replicate these findings in multiple regions of the triceps surae and investigate whether similar training strategies yield consistent effects in different muscle groups.

## 7 Practical Applications

The results of this study provide tentative support that initial partials may be a more effective method for promoting medial gastrocnemius hypertrophy than past‐failure partials in resistance‐trained individuals. However, past‐failure partials may still have utility as a supplementary method in scenarios where full‐ROM plantar flexion strength is required, with muscle hypertrophy as an additional benefit. Although these findings may extend to other muscle groups, additional research is needed to confirm broader applicability. Future studies should incorporate longer‐duration interventions and examine how these training strategies influence hypertrophy in other muscle groups.

## 8 Conclusion

The findings of this study support, albeit with limited evidential strength, the greater effectiveness of initial partials for promoting medial gastrocnemius hypertrophy. Although both training methods resulted in appreciable increases in muscle thickness, initial partials appeared to enhance hypertrophic adaptations to a greater extent. Further research is needed to confirm the robustness and generalizability of these findings.

## Conflicts of Interest

B.J.S. formerly served on the scientific advisory board for Tonal Corporation, a manufacturer of fitness equipment. No other authors report any declaration of interest.

## Supporting information

Supporting Information S1

Supporting Information S2

Supporting Information S3
