# Supplementary material for: Resistance Training Beyond Momentary Failure: The Effects of Past‐Failure Partials Versus Initial Partials on Calf Muscle Hypertrophy Among a Resistance‐Trained Cohort
Source: Eur J Sport Sci. 2025 Aug 24;25(9):e70030. doi: 10.1002/ejsc.70030 (PMC12375417; doi:10.1002/ejsc.70030)
Supplement: Supplementary file 1 — Supporting Information S1 [file EJSC-25-e70030-s003.pdf]

Supplementary file 1.

1.1.Standard method for assessment of resistance training in longitudinal design (SMART-LD) check-list for the items possible to try to reduce the chance for potential biases (Schoenfeld et al., 2023).

	Item	Description
**General**		
1.a)	The purpose of the study was clearly stated	Yes. The aim of this study was to compare the effect of initial partials versus past-failure partials in the standing calf raise exercise on medial gastrocnemius muscle hypertrophy after 8 weeks with resistance training among untrained men.
2.a)	The study was pre-registered prior to data collection for the outcomes of interest.	Yes. https://osf.io/f26u5
**Participant**		
3.a)	Sample size provided adequate statistical power or was appropriately justified.	Yes, we aimed to recruit as many participants as our resources would allow, to achieve the highest statistical power possible. We also utilized a within-participant design to further bolster power. We managed to recruit 26 participants.
4.a)	Inclusion/exclusion criteria were adequately identified.	Yes. Inclusion criteria were 1) age-range between 18-50 years, 2) trained in resistance training, which was defined as training twice a week for at least three years consistent prior to study start , 3) no previous self-reported use of anabolic steroids, 4) no musculoskeletal or cardiorespiratory disorders.
5.a)	Subject characteristics were clearly described.	Yes. Both mean and standard deviations for age, height, and body mass.
6.a)	Reasons for dropouts were adequately reported.	Yes. Consort flow chart is provided with dropouts and reasons.
7.a)	The study must report attendance and the mean participation must be ≥90% of the total number of sessions provided in the program.	Subjects who failed to participate at least 85% of training sessions (<14 sessions) were excluded from the analyses. Mean attendance was 15.2 sessions.
**Program**		
8.a)	Training program was written with sufficient detail so that the procedures can be replicated.	Yes. See below.
9.a)	Participants were randomly allocated between groups.	Yes. We randomized the right and left limb with www.randomizer.org to one out of the two conditions before the study.

10.a)	Randomization was concealed from investigators and participants.	Yes. Randomization of limbs was concealed from investigators and participants prior to study.
11.a)	Training was directly supervised.	Yes. Experienced personal trainers supervised all RT sessions.

	**Outcomes**	
12.a)	Assessments were written with sufficient detail so that the procedures can be replicated.	Yes. See methods.
13.a)	The primary outcome(s) were blinded to investigators.	No. This was not possible due to resource constraints. The same investigators supervised all RT sessions.
14.a)	Assessments employed validated methods for the purpose of the primary outcomes.	Yes. muscle thickness measurement with ultrasound imaging was used (Echo Wave 2 Software; Telemed, Latvia). A 60-mm probe size with 9 MHz scanning frequency, and Chemolan transmission gel (Chemodis, DA Alkmaar, The Netherlands).
15.a)	Proper preparation was employed for assessment methods where applicable.	Yes. Participant preparation: The subjects were instructed to not engage in any type of physical activity or training 72 hours before the pre- and post-test.
16.a)	Test-retest reliability measures were reported for assessments of the primary outcome(s) where applicable.	Yes. CV and TE values between the two pre-tests and post-tests are reported in the methods section.
	**Statistics**	
17.a)	Statistical analyses were written with sufficient detail so that the procedures can be replicated.	Yes. See statistics.
18.a)	Appropriate statistical tests were used for outcomes where applicable.	Yes. See statistics
19.a)	Pre- and post-study means, and variability and/or confidence intervals, were reported for all conditions in primary outcomes.	Yes. See statistics and results.
20.a)	Exact values were provided for reported statistics.	Yes. See results.
	**Final grading: 19/20 points.**	

1.2.RT protocol 1 and 2 used in the study.

RT1	Exercise	Sets	Repetitions	Intensity	Rest interval	Progression method	Note
A1	Lateral raises dumbbell	4 (week 1)<br>5 (week 2-8)	12-16	Momentary failure	30 seconds to a2	Double progression (12-16)	Start with a1 week 1, 3, 5, and 7.
A2	Lateral raises cable	4 (week 1)<br>5 (week 2-8)	12-16	Momentary failure	90 seconds to a1	Double progression (12-16)	Start with a2 week 2, 4, 6, and 8
B1	Leg presses 110 degrees knee flexion	3 (week 1)<br>4 (week 2-8)	8-12	Momentary failure	30 seconds to b2	Double progression (8-12)	Start with b1 week 1, 3, 5, and 7.
B2	Leg presses peak knee flexion	3 (week 1)<br>4 (week 2-8)	8-12	Momentary failure	120 seconds to b1	Double progression (8-12)	Start with b2 week 2, 4, 6, and 8
C1	Standing calf raises lengthened partials	3 (week 1)<br>4 (week 2-8)	10-20	Momentary failure	30 seconds to c2	Double progression (10-20)	Start with c1 week 1, 3, 5, and 7.
C2	Standing calf raises full repetitions + past-failure partials	3 (week 1)<br>4 (week 2-8	5+10, 5+10	Momentary failure	120 seconds to c1	Double progression (10-20)	Start with c2 week 2, 4, 6, and 8
**RT2**	**Exercise**	**Sets**	**Repetitions**	**Intensity**	**Rest pause**	**Progression method**	**Note**
A1	Standing calf raises lengthened partials	3 (week 1)<br>4 (week 2-8)	10-20	Momentary failure	30 seconds to c2	Double progression (10-20)	Start with a1 week 1, 3, 5, and 7.
A2	Standing calf raises full repetitions + past-failure partials	3 (week 1)<br>4 (week 2-8	5+10, 5+10	Momentary failure	120 seconds to c1	Double progression (10-20)	Start with a2 week 2, 4, 6, and 8
B1	Leg press peak 110 degrees knee flexion	3 (week 1)<br>4 (week 2-8)	8-12	Momentary failure	30 seconds to b2	Double progression (8-12)	Start with b1 week 1, 3, 5, and 7.
B2	Leg presses peak knee flexion	3 (week 1)<br>4 (week 2-8)	8-12	Momentary failure	120 seconds to b1	Double progression (8-12)	Start with b2 week 2, 4, 6, and 8
C1	Lateral raises dumbbell	4 (week 1)<br>5 (week 2-8)	12-16	Momentary failure	30 seconds to c2	Double progression (12-16)	Start with c1 week 1, 3, 5, and 7.
C2	Lateral raises cable	4 (week 1)<br>5 (week 2-8)	12-16	Momentary failure	90 seconds to c1	Double progression (12-16)	Start with c2 week 2, 4, 6, and 8

1.3.Alternative RT session.

Exercise	Sets	Repetitions	Repetitions in reserve	Rest interval	Progression method
Romanian deadlifts	2-3	8-12	1-3	120 seconds	Double progression (8-12)
Horizontal press exercise	2-3	8-12	1-3	120 seconds	Double progression (8-12)
Lat pull-down	2-3	8-12	1-3	120 seconds	Double progression (8-12)
Cable rows narrow grip	2-3	8-12	1-3	120 seconds	Double progression (8-12)
Bicep curls	2-3	8-12	1-3	120 seconds	Double progression (8-12)
Triceps extensions	2-3	8-12	1-3	120 seconds	Double progression (8-12)

The subjects were allowed to train the alernative RT session twice.

References

1) Schoenfeld, Androulakis-Korakakis, P., Coleman, M., Burke, R., & Piñero, A. (2023). SMART-LD: A tool for critically appraising risk of bias and reporting quality in longitudinal resistance training interventions.
